# Care Fragmentation Following Hospitalization for Atrial Fibrillation in the United States

**DOI:** 10.1016/j.jacadv.2023.100375

**Published:** 2023-06-07

**Authors:** Arjun Verma, Josef Madrigal, Troy Coaston, Nameer Ascandar, Catherine Williamson, Peyman Benharash

**Affiliations:** Cardiovascular Outcomes Research Laboratories, David Geffen School of Medicine at UCLA, Los Angeles, California, USA

**Keywords:** atrial fibrillation, care fragmentation, Nationwide Readmissions Database, nonelective readmissions, resource use

## Abstract

**Background:**

Despite the high prevalence of atrial fibrillation (AF), the incidence and impact of care fragmentation (CF) following hospitalization for this condition remain unstudied.

**Objectives:**

The present study used a national database to determine the incidence of and risk factors associated with CF. Outcomes following CF were also examined.

**Methods:**

All adults who were discharged alive following hospitalization for AF (index facility) were identified within the 2016 to 2019 Nationwide Readmissions Database. Patients requiring nonelective rehospitalization within 30 days of discharge were categorized into 2 groups. The CF cohort included those readmitted to a nonindex facility, while others were classified as noncare fragmentation. Multivariable regression was used to evaluate factors associated with CF, as well as its impact on in-hospital mortality, length of stay, and costs at rehospitalization.

**Results:**

Of an estimated 686,942 patients who met study criteria and survived to discharge, 13.6% (n = 93,376) experienced unplanned readmission within 30 days. Among those readmitted, 21.3% (n = 19,906) were readmitted to a nonindex facility. Patients who experienced CF were younger, more commonly male and less frequently readmitted for AF. Upon multivariable adjustment, male sex, Medicaid insurance (ref: private), and transfer status were associated with increased odds of CF. Upon readmission, CF was associated with a 18% increment in relative odds of in-hospital mortality, a 0.3-day increment in length of stay, and an additional $1,500 in hospitalization costs.

**Conclusions:**

CF was associated with significant clinical and financial burden. Further studies are needed to address factors which contribute to increased mortality and resource use following CF.

With increasing life expectancy and improved management of coexisting cardiac conditions, the prevalence of atrial fibrillation (AF) is projected to steadily rise.^1^ AF poses a major burden to patients and health care systems alike, accounting for nearly 500,000 hospitalizations in the United States each year.^2^ In addition, 1 in 7 patients require readmission within 2 weeks of discharge.^3^ A large body of literature has linked nonelective readmissions to greater mortality, complications and health care expenditures.^4^, ^5^, ^6^ Thus, programs such as the Hospital Readmission Reduction Program have enacted a multifaceted approach to curb unplanned readmissions.

Defined as readmission to a nonindex facility, care fragmentation (CF) has been associated with particularly poor outcomes. Prior investigations of CF, primarily in surgical patients, have cited a multitude of factors including the absence of universal medical records, variation in center expertise, and delays associated with repeat diagnostic testing as potential reasons for such findings.^7^, ^8^, ^9^ Furthermore, in a decade-long study of ∼217,000 Canadian patients with heart failure, CF was found to be associated with increased odds of death as well as greater hospitalization costs.^7^ Despite the high prevalence of AF and associated conditions, the incidence and impact of CF following hospitalization for this condition remain unstudied.

In the present work, we used a contemporary and nationally representative cohort of hospitalizations for AF to examine factors associated with 30-day readmission and CF. We further evaluated the risk-adjusted impact of CF on several clinical and financial endpoints, hypothesizing that CF would be associated with increased mortality and resource utilization upon rehospitalization.

## Methods

### Data source

We performed a retrospective cohort study using data from the 2016 to 2019 Nationwide Readmissions Database (NRD). Maintained by the Healthcare Cost and Utilization Project, the NRD is the largest, publicly available all-payer readmissions database and samples discharges from 28 geographically diverse states.^10^ Using robust survey weighting methodology, the NRD provides accurate estimates for approximately 60% of hospitalizations in the United States. Unique linkage numbers are used to track readmissions within each state and calendar year.

### Patient and public involvement

The Agency for Healthcare Research and Quality collected patient records. Patients were not involved in data collection, research questions, study design, or analysis. Patients did not serve as authors or contributors in the study. There are no plans to disseminate the study results to the patients. Due to the deidentified nature of the NRD, the present study was deemed exempt from full review by the Institutional Review Board at the University of California, Los Angeles.

### Study cohort

All nonelective adult (≥18 years) hospitalizations with a primary diagnosis of AF were identified using International Classification of Diseases-10th Revision (ICD-10) diagnosis codes (Supplemental Table 1). Patients admitted in December of each year were excluded to allow for 30 days of follow-up after discharge. In addition, those who underwent cardiothoracic operations or died during index hospitalization were not included for further study. Records with missing key variables such as age, sex, and mortality were excluded (1%).

### Variable definitions

Patients requiring nonelective rehospitalization within 30 days of index discharge were categorized into 2 groups: the CF cohort comprised patients who were readmitted to an institution other than the index, while others were classified as noncare fragmentation (nCF). Patient and hospital characteristics including age, sex, income level, insurance coverage, and hospital teaching status were defined in accordance with the NRD Data Dictionary.^10^ The Van Walraven modification of the Elixhauser Comorbidity Index, a validated composite of 30 comorbidities, was used to quantify the burden of chronic conditions.^11^ Individual comorbidities, such as congestive heart failure, coronary artery disease, liver disease, and end stage renal disease, were tabulated using ICD-10 diagnosis codes. Similarly, acute events were ascertained and grouped into neurologic (ischemic/hemorrhagic stroke), thrombotic (pulmonary embolism/deep vein thrombosis), respiratory (pneumonia, respiratory failure, pneumothorax), gastrointestinal (bowel ischemia/perforation, liver infarction, hemorrhagic necrosis, hepatic vein thrombosis) categories. Urinary tract infection and sepsis were considered as independent complications.^12^ Comorbidities and acute events were defined and analyzed with respect to the index hospitalization. Inpatient costs were generated via application of hospital specific cost-to-charge ratios and inflation adjusted to the 2019 Personal Health Index.^13^ To evaluate the indications for readmission, primary ICD-10 diagnosis codes were tabulated and categorized into clinically relevant groups.

### Outcomes

The primary study endpoint was CF. The association of CF with in-hospital mortality, length of stay (LOS), hospitalization costs, and discharge disposition at readmission was subsequently evaluated.

### Statistical analysis

Categorical variables are reported as proportions and compared across groups using the Pearson chi-square test. Continuous variables are summarized as medians with IQR and analyzed with the Mann-Whitney *U* test. Multivariable logistic and linear regressions were fit to estimate the risk-adjusted association between various factors and outcomes of interest. Regressions for LOS and costs were fit after exclusion of records in the top 95th percentile, given the skewed distributions of these variables. Variable selection was performed via application of elastic net regularization, a technique that combines the Least Absolute Shrinkage and Selection Operator and Ridge regression penalties to reduce collinearity and external bias. Final models were determined using the area under the received operating characteristic (C-statistic) and coefficient of determination (R^2^) for logistic and linear regression, respectively. Regression outputs are reported as adjusted odds ratios (AORs) or β-coefficients with 95% CIs. All statistical analysis was performed using Stata 16.1 (StataCorp).

## Results

Of an estimated 686,942 patients who met study criteria and survived to discharge, 13.6% (n = 93,376) experienced unplanned readmission within 30-days. Compared to their counterparts, readmitted patients were older (75 [IQR: 66-83] years vs 72 [IQR: 63-81] years, *P* < 0.001), more commonly female (56.3%, n = 52,601/93,376 vs 52.7%, n = 312,571/593,566, *P* < 0.001) and had a greater median Elixhauser Comorbidity Index (5 [IQR: 4-7] vs 4 [IQR: 3-6], *P* < 0.001). Notably, readmitted patients more commonly carried Medicare (79.8%, n = 74,489/93,376 vs 70.5%, n = 418,352/593,566, *P* < 0.001) and Medicaid (6.4%, n = 5,961/93,376 vs 5.2%, n = 30,743/593,566, *P* < 0.001) insurance than nonreadmitted patients. Those who experienced 30-day readmission were also more frequently transferred from another hospital to the index facility (1.7%, n = 1,606/93,376 vs 1.2%, n = 7,182/591,516, *P* < 0.001), compared to patients not readmitted within 30 days. Comparison of additional index hospitalization characteristics between readmitted and nonreadmitted patients are displayed in Table 1.

**Table 1:** Index Hospitalization Characteristics of Patients Who Survived to Discharge, Stratified by 30-Day, Nonelective Readmission

	Not Readmitted (n = 593,566)	30-d Readmission (n = 93,376)	*P* Value
Age (y)	72 (63-81)	75 (66-83)	<0.001
Female	52.7	56.3	<0.001
Transfer into index facility	1.2	1.7	<0.001
Income level (%, percentile)			<0.001
76th-100th	20.0	18.3	
51st-75th	25.7	24.2	
26th-50th	27.9	28.6	
1st-25th	26.4	28.8	
Insurance type			<0.001
Private	20.1	10.6	
Medicare	70.5	79.8	
Medicaid	5.2	6.4	
Other	4.3	3.3	
Elixhauser Comorbidity Index	4 (3-6)	5 (4-7)	<0.001
Comorbidities			
Congestive heart failure	41.1	55.9	<0.001
Coronary artery disease	35.2	43.1	<0.001
Valve disease	21.9	26.2	<0.001
Pulmonary circulation disorder	8.9	13.4	<0.001
Peripheral vascular disease	10.0	13.3	<0.001
Neurologic disorder	5.2	7.4	<0.001
Hypothyroidism	19.3	21.5	<0.001
End stage renal disease	2.6	6.2	<0.001
Liver disease	2.9	4.5	<0.001
Coagulopathy	4.7	6.8	<0.001
Weight loss	3.3	5.9	<0.001
Electrolyte imbalance	25.3	33.6	<0.001
Anemia	3.8	6.0	<0.001
Electrical cardioversion	19.2	16.4	<0.001
Catheter ablation	0.2	0.2	0.90
Hospital characteristics			
Bed size			0.08
Large	53.1	53.8	
Medium	29.4	29.0	
Small	17.5	17.2	
Location and teaching status			0.82
Rural	9.6	9.6	
Metropolitan, nonteaching	25.1	25.3	
Metropolitan, teaching	65.2	65.1	

Values are median (IQR) or % unless otherwise indicated.

**Figure 1:**
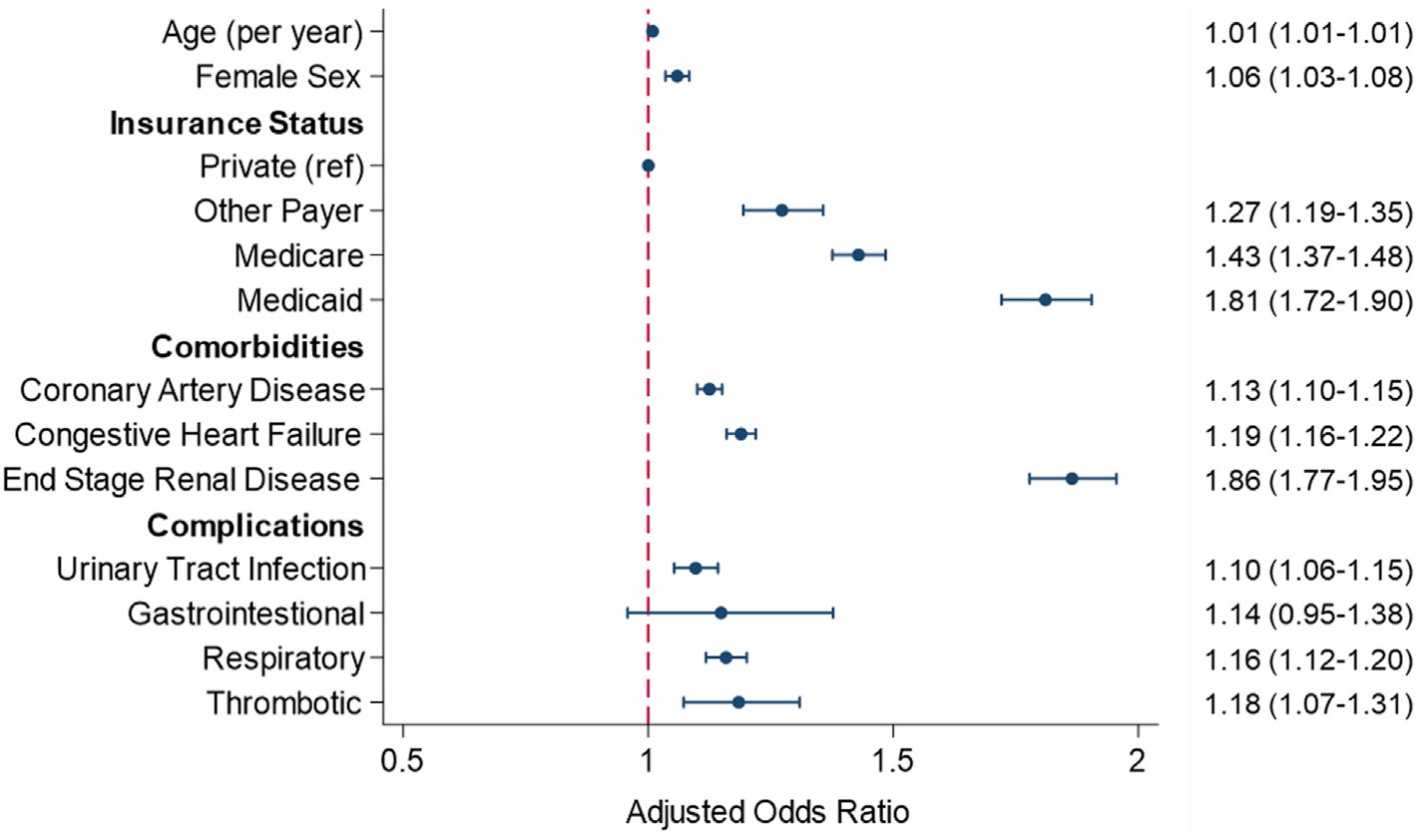
**Patient Factors Associated With Odds of 30-Day Readmission** Displayed estimates are obtained from multivariable logistic regression model with 30-day, nonelective readmission as the dependent variable. C-statistic for the multivariable logistic regression model 0.66.

Upon multivariable adjustment (C-statistic 0.66), advanced age, female sex, and higher Elixhauser Comorbidity Index were associated with greater odds of readmission (Figure 1). Individual chronic conditions linked to readmission included congestive heart failure, coronary artery disease and end stage renal dysfunction (Supplemental Table 2). Relative to private, Medicare (AOR: 1.43; 95% CI: 1.37-1.48) and Medicaid (AOR: 1.81; 95% CI: 1.72-1.90) insurance were associated with significantly increased odds of readmission. Patients who were transferred into the index hospital had a 14% reduction in relative odds of readmission (AOR: 0.86; 95% CI: 0.79-0.94). Although neurologic complications at index hospitalization did not alter odds of readmission, patients who developed thrombotic or respiratory complications during the index admission had greater risk (Figure 1). Patients who experienced nonhome discharge had increased odds of readmission, compared to those who were discharged to home after the index hospitalization (AOR: 1.18; 95% CI: 1.14-1.22).

**Figure 2:**
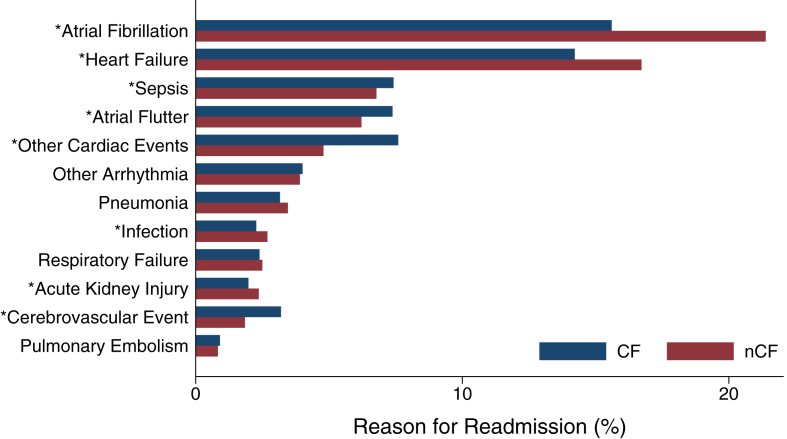
**Indications for Readmission by CF Status** ∗*P* < 0.05. CF = care fragmentation; nCF = noncare fragmentation.

Of those readmitted within 30 days, 21.3% (n = 19,906) experienced CF. Compared to nCF, the CF cohort was slightly younger (74 [IQR: 64-82] years vs 75 [IQR: 66-83] years, *P* < 0.001) but less commonly female (52.4%, n = 10,431/19,906 vs 57.4%, n = 42,169/73,470, *P* < 0.001). Patients who experienced CF were less commonly readmitted for AF and heart failure, but more frequently for cerebrovascular events, relative to nCF (Figure 2). The rate of sepsis was greater among CF patients, while the incidence of pneumonia, respiratory failure, and pulmonary embolism were comparable between groups. The CF cohort was more commonly insured by Medicaid (8.1%, n = 1,608/19,906 vs 5.9%, n = 4,353/73,470, *P* < 0.001) and more likely to have been transferred into the index hospital (4.3%, n = 851/19,906 vs 1.0%, n = 755/73,470, *P* < 0.001), compared to nCF. In addition, patients who experienced CF more commonly underwent index admission at hospitals with small bed size and rural location, relative to nCF (Table 2). A comprehensive comparison of patient characteristics and outcomes between the CF and nCF cohorts is shown in Table 2.

**Table 2:** Comparison of Index Hospitalization Characteristics Between the CF and nCF Cohort

	nCF (n = 73,470)	CF (n = 19,906)	*P* Value
Age (y)	75 (66-83)	74 (64-82)	<0.001
Female	57.4	52.4	<0.001
Transfer into index facility	1.0	4.3	<0.001
Income level (%, percentile)			<0.001
76th-100th	18.5	17.7	
51st-75th	24.7	22.6	
26th-50th	28.5	29.1	
1st-25th	28.4	30.5	
Insurance type (%)			<0.001
Private	10.5	10.7	
Medicare	80.5	77.2	
Medicaid	5.9	8.1	
Other	3.1	3.9	
Elixhauser Comorbidity Index	5 [4-7]	5 [4-7]	0.32
Comorbidities			
Congestive heart failure	56.1	55.4	0.27
Coronary artery disease	43.2	42.7	0.33
Valve disease	26.5	25.2	0.016
Pulmonary circulation disorder	13.5	13.0	0.21
Peripheral vascular disease	13.3	13.4	0.67
Neurologic disorder	7.2	8.0	0.004
Hypothyroidism	22.0	19.8	<0.001
End stage renal disease	6.1	6.5	0.15
Liver disease	4.3	4.9	0.012
Coagulopathy	6.7	7.1	0.20
Weight loss	6.0	5.8	0.59
Electrolyte imbalance	33.4	34.4	0.07
Anemia	6.0	6.0	0.86
Electrical cardioversion	7.7	8.0	0.047
Catheter ablation	0.3	0.2	<0.001
Hospital characteristics			
Bed Size			<0.001
Large	55.8	46.4	
Medium	28.6	30.6	
Small	15.6	23.0	
Location and teaching status			<0.001
Rural	9.2	11.3	
Metropolitan, nonteaching	24.3	28.8	
Metropolitan, teaching	66.5	59.9	

Values are median (IQR) or % unless otherwise indicated.

CF = care fragmentation cohort; nCF = noncare fragmentation cohort.

**Figure 3:**
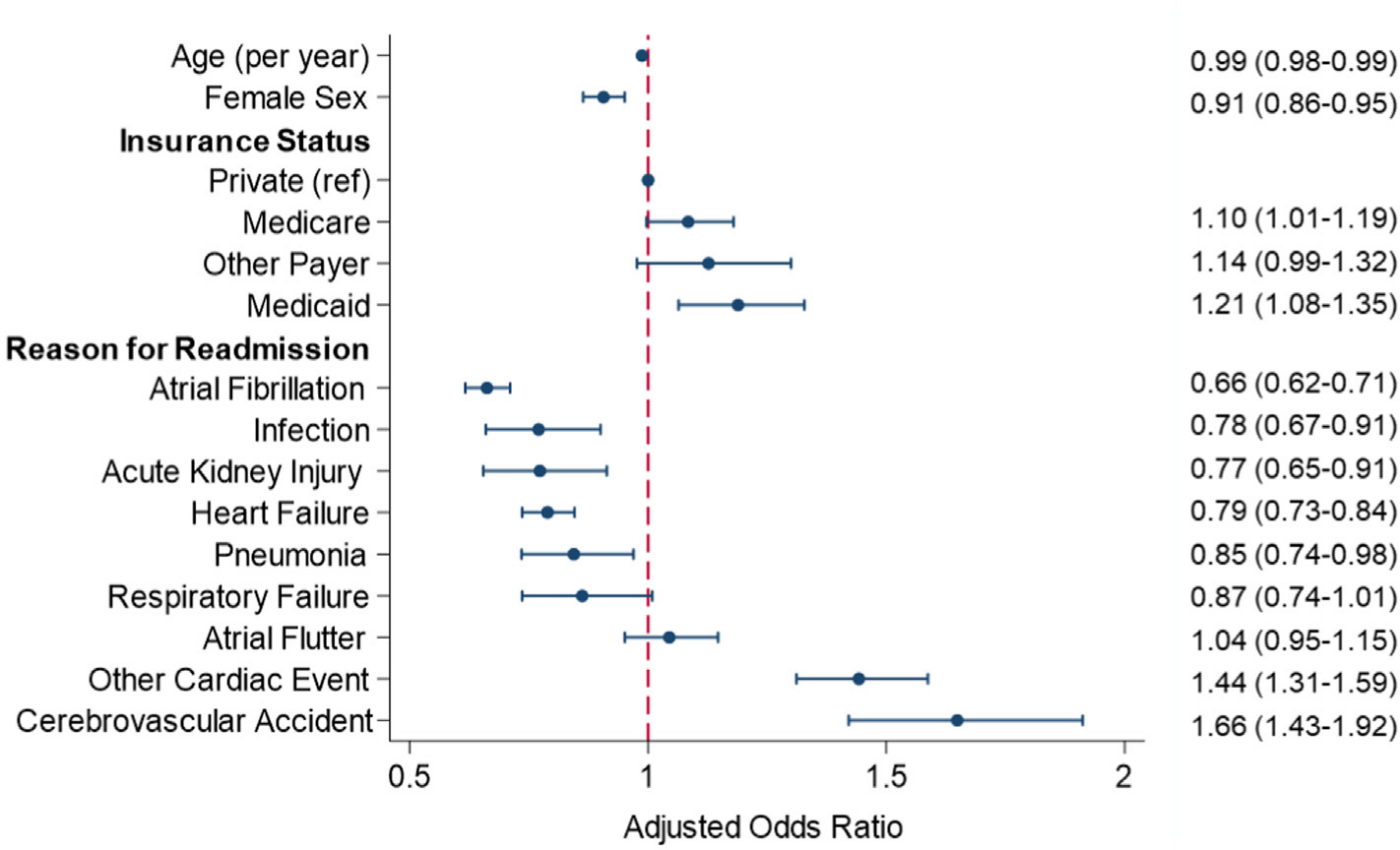
**Patient Factors Associated With CF** Displayed estimates are obtained from multivariable logistic regression model with care fragmentation as the dependent variable. C-statistic for the multivariable logistic regression model 0.60. CF = care fragmentation.

We developed a logistic regression model to identify factors independently associated with CF (Supplemental Table 3). Interestingly, increasing age and female sex were linked to decreased odds of CF (Figure 3). Patients who were readmitted for AF (AOR: 0.66; 95% CI: 0.62-0.71) or heart failure (AOR: 0.79; 95% CI: 0.73-0.84) had reduced odds of CF, while those with neurologic and other cardiac events had greater odds (Figure 3). Patients with Medicaid coverage experienced a 21% increase in relative odds of CF (ref: private). Those who were transferred experienced a 4.6-fold increment in odds of CF. Although acute events at index hospitalization did not influence the odds of CF, patients who were discharged to skilled nursing facilities had greater odds (AOR: 1.49; 95% CI: 1.40-1.59).

**Central Illustration:**
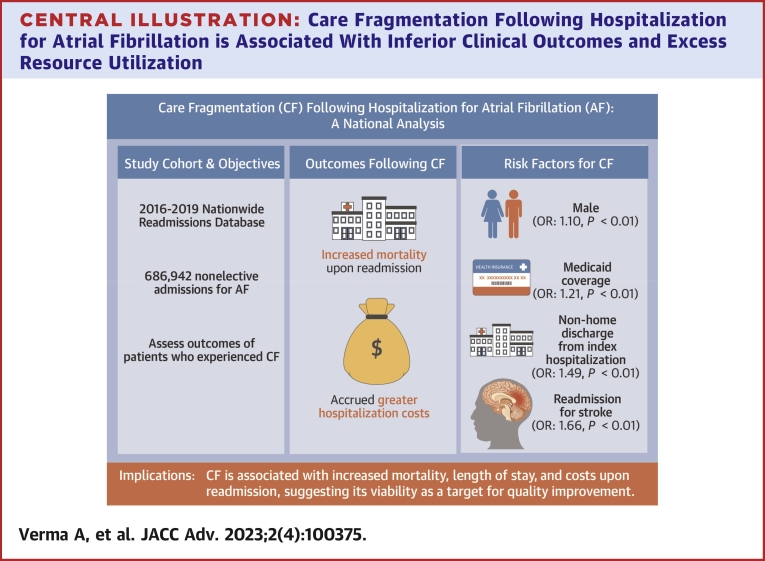
Care Fragmentation Following Hospitalization for Atrial Fibrillation is Associated With Inferior Clinical Outcomes and Excess Resource Utilization

A bivariate comparison of outcomes between the CF and nCF cohorts is shown in Table 3. Following adjustment for several patient factors including readmission etiology, CF was associated with significantly increased odds of in-hospital mortality during readmission (AOR: 1.18; 95% CI: 1.06-1.32). Of note, fragmented care was associated with a 0.3-day increment in LOS and an additional $1,500 in hospitalization costs (Table 3).

**Table 3:** Adjusted Associations Between CF and Outcomes of Interest, Relative to nCF

	nCF (n = 73,470)	CF (n = 19,906)	Unadjusted OR (95% CI)	*P* Value	Adjusted OR (95% CI)	*P* Value
In-hospital mortality	4.5	5.5	1.24 (1.12-1.37)	<0.001	1.18 (1.06-1.32)	0.003
Length of stay (d)	4 (2-6)	4 (2-8)	1.1 (1.0-1.3)	<0.001	0.3 (0.2-0.4)	<0.001
Hospitalization costs ($1,000s)	7.7 (4.6-14.2)	9.7 (5.5-19.1)	4.4 (3.9-4.9)	<0.001	1.5 (1.3-1.8)	<0.001
Nonhome discharge	24.7	28.1	1.19 (1.13-1.25)	<0.001	1.13 (1.06-1.21)	<0.001

Values are % or median (IQR) unless otherwise indicated.

CF = care fragmentation cohort; nCF = noncare fragmentation cohort.

## Discussion

While quality improvement programs such as the Hospital Readmission Reduction Program aim to reduce medical and surgical readmissions, specific policies targeting CF are lacking.^14^ In this national analysis of AF hospitalizations, we found that 1 in 5 patients readmitted within 30 days of discharge experienced CF and returned to a nonindex facility. At readmission, patients who experienced CF had increased risk of mortality, experienced longer LOS and accrued greater costs. In our multivariable regression model, several patient characteristics associated with greater odds of CF were identified, including Medicaid coverage, transfer status, and indication for readmission.

Prior studies have found CF to be associated with poor outcomes at readmission following major operations and heart failure hospitalizations.^7^^,^^15^^,^^16^ In congruence with these reports, the present study demonstrated that CF was associated with a nearly 20% increment in mortality. Although the reasons for this finding are multifactorial, inadequate health information transfer between centers is likely a major driver of poor outcomes observed among patients readmitted to nonindex hospitals. Some have previously demonstrated information discontinuity to result in diagnosis and treatment delays, worsening patient outcomes.^9^^,^^17^^,^^18^ Moreover, the detrimental consequences of ineffective information transfer between providers is likely amplified in the setting of AF, as these patients often have complex treatment plans and multimorbidity.^19^, ^20^, ^21^ Thus, care teams at nonindex hospitals may benefit from interhospital health information exchange systems to inform decision-making and deliver more efficient care.^9^^,^^18^^,^^22^ Given the limitations of national studies, analyses of factors that are responsible for the poor outcomes associated with CF in more granular datasets are needed.

Importantly, we found that patients readmitted to a nonindex institution faced prolonged LOS and a $1,500 increase in costs, compared to those readmitted to the originating facility. This amounts to nearly $30,000,000 in expenditures attributable to CF each year. As such, although readmissions are recognized contributors to health care expenditures, our findings suggest that some rehospitalizations, specifically those to nonindex hospitals, disproportionately burden the health care system. The most frequently cited indication for high resource use in fragmented readmissions is the duplication of costly tests, procedures, and treatments.^7^^,^^17^ Additionally, in the outpatient setting, Frandsen et al examined CF and attributed higher costs to uncoordinated consultation with various physicians, which may be particularly relevant to patients affected by chronic conditions such as AF.^16^^,^^23^ Our findings highlight the extensive financial burden of CF, which may be ameliorated by the adoption of universal medical record systems.^9^^,^^18^ Nonetheless, the development and dissemination of novel cost mitigation strategies may also align with goals of value-based health care delivery.

Using multivariable regression, we identified several patient characteristics that were associated with CF. Notably, we found that Medicaid insurance was associated with a 1.21-fold increase in odds of readmission to a nonindex institution. Our finding is congruent with several reports that cite Medicaid coverage as a factor linked to CF after major operations as well as medical hospitalizations.^24^, ^25^, ^26^, ^27^, ^28^ Moreover, a survey of patients with chronic conditions found Medicaid enrollment to be associated with fragmented care, primarily due to reduced access to health services that accept this insurance.^24^^,^^25^ This finding highlights that social determinants of health may affect CF and indicate a possible pathway for improvement. In particular, more rigorous predischarge counseling and efforts by the index hospital to coordinate follow up visits may mitigate the detrimental clinical effects of CF.^26^ In addition to insurance, transfer status was also noted to be positively associated with CF in this population. The current literature has cited transportation and geographic barriers in returning to the receiving institution to contribute to increased CF in this vulnerable cohort. While not measurable in the present study, distance to the index facility has also been associated with greater CF. Taken together, our findings may inform efforts to develop strategies that aim to mitigate CF following hospitalization for AF.

### Study Limitations

The present study has several important limitations. Inherent to the retrospective study design, we are unable to draw causative conclusions. In addition, due to the administrative nature of the NRD, we do not have access to granular clinical information including medication regiment and ejection fraction as well as echocardiographic and electrocardiographic findings. Importantly, our reliance on ICD-10 diagnosis and procedure codes prevents us from ascertaining the exact reason for which a patient was readmitted. Moreover, whether urgency of readmission or lack of access to the index facility contributed to rehospitalization at a nonindex institution is not discernable. Indeed, it is possible that some patients experienced CF due to need for advanced therapies (catheterization laboratories) that were not present at the index hospital. We were also unable to evaluate the mode through which patients were readmitted (self-present, ambulance, etc). Despite such limitations, the NRD is uniquely able to capture nonindex readmission at the large scale.

## Conclusions

Approximately 1 in 5 patients who were readmitted following an AF hospitalization experienced CF (presenting to a nonindex institution). Such patients had higher risk of mortality and accrued greater health care expenditure (Central Illustration).

## Funding support and author disclosures

The authors have reported that they have no relationships relevant to the contents of this paper to disclose.

PERSPECTIVES **COMPETENCY IN PATIENT CARE:** Targeted postdischarge care pathways for patients at high risk for CF may reduce the associated clinical and financial burden.

**COMPETENCY IN SYSTEMS BASED PRACTICE:** CF may represent one reason why publicly insured patients face inferior long-term outcomes and increased health care expenditure.

**TRANSLATIONAL OUTLOOK:** Further studies using granular databases are necessary to identify strategies that can be implemented to mitigate care fragmentation as well as its associated clinical and financial burden.
