# Supplementary material for: Care Fragmentation Following Hospitalization for Atrial Fibrillation in the United States
Source: JACC Adv. 2023 Jun 7;2(4):100375. doi: 10.1016/j.jacadv.2023.100375 (PMC11198211; doi:10.1016/j.jacadv.2023.100375)
Supplement: Supplemental Tables 1-3 [file mmc1.pdf]

Supplemental Table 1: *International Classification of Diseases 10th Revision* diagnosis codes used to identify patients with atrial fibrillation.

**Condition**	ICD-10 Diagnosis Code
Atrial Fibrillation	I480, I483, I484<br>I481, I4811, I4812<br>I482, I4821, I4822<br>I489, I4891, I4892

Supplemental Table 2: Full multivariable logistic regression for 30-day non-elective readmission following hospitalization for atrial fibrillation.

Parameter	Adjusted Odds Ratio	95% Confidence Interval	P-Value
Age (per year)	1.01	1.01-1.01	<0.001
Female Sex	1.06	1.03-1.08	<0.001
Transfer to Index Facility	0.86	0.79-0.94	0.001
Income Level (Percentile)			
76th-100th	ref		
51st-75th	0.99	0.96-1.03	0.71
26th-50th	1.04	1.01-1.08	0.011
1st-25th	1.06	1.03-1.10	<0.001
Insurance Type			
Private	ref		
Medicare	1.43	1.37-1.48	<0.001
Medicaid	1.81	1.72-1.90	<0.001
Other Payer	1.27	1.19-1.35	<0.001
**Comorbidities**			
Elixhauser Comorbidity Index (per point)	1.15	1.14-1.16	<0.001
End Stage Renal Disease	1.86	1.77-1.95	<0.001
Congestive Heart Failure	1.19	1.16-1.22	<0.001
Coronary Artery Disease	1.13	1.10-1.15	<0.001
Valve Disease	0.93	0.91-0.96	<0.001
Pulmonary Circulation Disorder	0.95	0.92-0.98	0.005
Peripheral Vascular Disease	0.98	0.95-1.02	0.37
Chronic Lung Disease	1.20	1.17-1.23	<0.001
Hypothyroidism	0.90	0.88-0.93	<0.001
Liver Disease	1.11	1.04-1.17	0.001
Coagulopathy	0.99	0.95-1.04	0.65
Weight Loss	1.11	1.06-1.18	<0.001
Electrolyte Imbalance	1.03	1.01-1.06	0.010
Anemia	1.05	1.00-1.10	0.037
**Acute Events at Index Admission**			
Neurologic	0.98	0.89-1.09	0.72
Thrombotic	1.18	1.07-1.31	0.001
Respiratory	1.16	1.12-1.20	<0.001
Gastrointestinal	1.14	0.95-1.37	0.17
Urinary Tract Infection	1.10	1.06-1.15	<0.001
Sepsis	0.83	0.73-0.94	0.003
**Resource Utilization at Index Admission**			
Length of Stay (per day)	1.03	1.02-1.03	<0.001

Hospitalization Costs (per $1,000)	0.99	0.99-0.99	<0.001
Non-Home Discharge	1.18	1.14-1.22	<0.001

Supplemental Table 3: Full multivariable logistic regression model for care fragmentation following hospitalization for atrial fibrillation.

Parameter	Adjusted Odds Ratio	95% Confidence Interval	P-Value
Age (per year)	0.99	0.98-0.99	<0.001
Female Sex	0.91	0.86-0.95	<0.001
Transfer into Index Facility	4.60	3.95-5.35	<0.001
Income Level (Percentile)			
76th-100th	ref		
51st-75th	0.95	0.88-1.03	0.20
26th-50th	1.05	0.97-1.13	0.20
1st-25th	1.06	0.98-1.15	0.13
Insurance Type			
Private	ref		
Medicare	1.10	1.01-1.19	0.035
Medicaid	1.21	1.08-1.35	0.001
Other Payer	1.14	0.99-1.32	0.07
**Comorbidities**			
Peripheral Vascular Disease	1.00	0.93-1.07	0.99
Neurologic Disorder	1.01	0.93-1.10	0.83
Chronic Lung Disease	0.91	0.87-0.96	<0.001
Hypothyroidism	0.96	0.91-1.02	0.22
Weight Loss	0.92	0.83-1.02	0.11
Electrolyte Imbalance	1.02	0.97-1.07	0.49
**Acute Events at Index Admission**			
Neurologic	1.14	0.93-1.41	0.21
Respiratory	1.05	0.98-1.13	0.19
Urinary Tract Infection	0.98	0.89-1.07	0.61
Sepsis	1.04	0.83-1.30	0.75
**Resource Utilization at Index Admission**			
Length of Stay (per day)	0.99	0.98-1.00	0.039
Hospitalization Costs (per $1,000 USD)	1.00	1.00-1.00	0.92
Non-Home Discharge	1.49	1.40-1.59	<0.001
**Reason for Readmission**			
Cerebrovascular Accident	1.66	1.43-1.92	<0.001
Other Cardiac Event	1.44	1.31-1.59	<0.001
Atrial Flutter	1.04	0.95-1.15	0.37
Respiratory Failure	0.87	0.74-1.01	0.07
Pneumonia	0.85	0.74-0.98	0.025
Heart Failure	0.79	0.73-0.84	<0.001
Acute Kidney Injury	0.77	0.65-0.91	0.002
Infection	0.78	0.67-0.91	0.002
Atrial Fibrillation	0.66	0.62-0.71	<0.001
